# Social contributions to meaning in life: the role of romantic relationship quality, parenting, and gender

**DOI:** 10.3389/fpsyg.2024.1349642

**Published:** 2024-02-08

**Authors:** Alaina I. Gold, Yana Ryjova, Elizabeth C. Aviv, Geoffrey W. Corner, Hannah F. Rasmussen, Yehsong Kim, Gayla Margolin

**Affiliations:** ^1^Department of Psychology, Dornsife College of Letters, Arts and Sciences, University of Southern California, Los Angeles, CA, United States; ^2^VA Puget Sound Health Care System, Veterans Health Administration, United States Department of Veterans Affairs, Seattle, WA, United States

**Keywords:** meaning in life, romantic relationships, relationship quality, parenthood, COVID

## Abstract

**Introduction:**

The present study tests the association between romantic relationship quality and number of children on meaning in life (i.e., sense of purpose, coherence, and significance) and considers interactions between these constructs and gender.

**Methods:**

A survey was conducted approximately one year into the pandemic among 473 individuals in the United States.

**Results:**

Models demonstrated that relationship quality and number of children are positively associated with meaning, though relationship quality was more strongly related to meaning for men than women. We showed that for women there was an equally positive link between relationship quality and meaning regardless of number of children. However, for men, the positive association between relationship quality and meaning was strongest for those with more than one child, decreased in magnitude for those with one child, and was no longer significant for men with more than one child.

**Discussion:**

These findings provide empirical evidence that social relationships benefit meaning in life and underscore the complexity of these associations. Results have implications for theoretical perspectives on meaning in life, as well as for policies that encourage family wellbeing.

## Introduction

Social connections define and shape the human experience. A hallmark of our species is the complexity and salience of our social relationships, and almost all of life’s meaningful moments involve important others. Whether celebrating the birth of a child, marrying a life partner, caring for an ailing loved one, or simply living through the day-to-day pleasures of social interactions, the experience of meaning in life is most often found in and created through interdependence with others. Research converges with the lay understanding that social relationships serve as a primary source of meaning in life (Baumeister, 1991; Debats, 1999; Lambert et al., 2013). The current study extends that research by examining familial sources of meaning in life, including romantic relationship quality and parenthood.

Meaning in life, or ‘the extent to which people comprehend, make sense of, or see significance in their lives, accompanied by the degree to which they perceive themselves to have a purpose, mission, or overarching aim in life’ (Steger, 2009, p. 682) plays a central role in individuals’ well-being. Meaning represents the degree to which an individual views their life as purposeful, coherent, and significant beyond day-to-day suffering. Several factors contribute to meaning in life, including social connection, positive affect, religious and global worldviews, and sense of self (for a review see King and Hicks, 2021). Among these factors, social support (Golovchanova et al., 2021) and connection seem to play a particularly important bidirectional role (Stavrova and Luhmann, 2016). Previous research has found that a sense of belonging (e.g., Lambert et al., 2013) or connectedness (Chen et al., 2022) are two socially grounded feelings that enhance meaning in life. Based on daily diary data, days in which positive social events occur are perceived by individuals as most meaningful (Machell et al., 2015). The quality of social connection is also related to aspects of meaning in life, such that people feel a greater sense of purpose on days with better social interactions (Pfund et al., 2022) and when relationships have higher support and lower strain (Weston et al., 2020). Conversely, individuals who experience social exclusion (Stillman et al., 2009) or interpersonal rejection (Twenge et al., 2003) perceive life as less meaningful.

Any social relationship that enhances feelings of belonging and connectedness are likely to support an individual’s sense of meaning (Stavrova and Luhmann, 2016). More specifically, familial relationships are consistently reported to be the single most significant contributor to meaning in life (Lambert et al., 2010; Glaw et al., 2017). Romantic relationships may be a particularly salient context in which people develop a sense of meaning in life. For example, individuals find greater meaning when spending time with a spouse (Flood and Genadek, 2016), and those who regularly forgive their romantic partners report increased meaning in life over time (Van Tongeren et al., 2015). Although these studies demonstrate romantic relationships are positively linked to meaning, very few studies have examined whether the quality of the relationship plays a distinct role. Only one study, in a sample of Israeli first-time mothers both pre-and during-COVID-19, has shown that higher marital quality was linked to greater meaning in life (Chasson et al., 2021). Related work, also in a sample of women across the transition to parenthood, suggests relationship quality is an important longitudinal predictor of life satisfaction (Dyrdal et al., 2011). More studies have focused on the effect in reverse (i.e., meaning predicting relationships). For instance, higher meaning in life has predicted global measures of better relationship functioning, such as greater relationship satisfaction (Hadden and Knee, 2018; Yu and Chang, 2021). An increased sense of purpose—one facet of meaning in life—is associated with a higher incidence of marriage (Pinquart, 2002), greater relationship commitment, and the perception that one’s partner is preferable to alternatives (Pfund et al., 2020).

A small body of research that has investigated the impact of parenthood on meaning in life suggests that the parenting role can confer enhanced meaning, in part due to positive impacts on a sense of purpose in life (Hughes, 2006). For example, parents spend more time thinking about meaning than non-parents, and parents find more meaning in life when taking care of their children compared to other daily activities (Nelson et al., 2013). Additionally, time spent with one’s children can increase feelings of meaning and purpose in life, as parents derive a sense of fulfillment from the ongoing processes of raising their children (Baumeister et al., 2013; Musick et al., 2016). Furthermore, higher levels of meaning in life predicted greater well-being for parents but not for non-parents in a U.S. sample (Nelson et al., 2013). Thus, parenthood can provide a sense of direction and purpose in individuals’ lives (Nelson et al., 2014) and having multiple children may increase the amount of opportunities for a sense of belonging or purpose. At the same time, there is also an inverse relationship between parenting stress and meaning in life (Taubman-Ben-Ari et al., 2021) and, for women only, children beyond the first child decrease subjective well-being (Kohler, 2012). Becoming a parent, or having additional children, involves a role shift in which relationships and identities are modified to make room for the new baby. This change in roles comes with increased stress and responsibility (Vismara et al., 2016), which could have downstream impacts on meaning in life (Park, 2010). Taken together, the existing literature supports the notion that the day-to-day experience of parenting impacts meaning in life.

Less is known about the role of gender in the association between social relationships and meaning in life. Some research indicates that relationships are a more significant source of meaning for women than for men (Debats, 1999; Hadden and Knee, 2018). This notion is supported, in part, by reports that women experience less social isolation (Röhr et al., 2022) and possess more confidants in their social networks (McPherson et al., 2006). However, recent studies have shown that gender does not moderate the contribution of a sense of purpose to relationship quality or commitment (Pfund et al., 2020). Additional work has investigated the role of gender in the association between meaning in life and parenting (e.g., Goodman et al., 2019; Corner et al., 2023) and found that the association between family relationships and meaning in life was stronger for women than for men. Other work indicates that, while specific parenting responsibilities do differ by gender, meaning derived from parenting does not (Musick et al., 2016). Yet, recent research suggests that parenting is associated with greater overall indicators of well-being for fathers compared to mothers (Schieman and Taylor, 2001; Nelson-Coffey et al., 2019). Given differences in past research with regard to gender, it is clear that romantic relationships and parenting are relevant to meaning for men and women and it is imperative to understand any differences in these patterns. Thus, our study fills an important gap by exploring gender as a moderator.

Finally, very few existing studies have investigated the interplay between multiple forms of social connection on meaning in life. This is important because the different roles that people play (e.g., partner, parent) can be different avenues by which to derive meaning in life. In particular, to our knowledge, no study has examined the interaction between romantic relationship functioning and the number of children in the home. Extensive research has documented a decline in relationship satisfaction across the transition to parenthood (e.g., Huss and Pollmann-Schult, 2020), but less is known about whether parenthood may similarly impact the association between relationship quality and meaning in life. Related work found that parents who experienced a greater sense of meaning during their childbirth actually endorsed smaller declines in relationship satisfaction across the transition to parenthood, particularly for mothers (Corner et al., 2023). Furthermore, though parenting may reduce relationship satisfaction, it can improve factors of relationship functioning like relationship commitment (Huss and Pollmann-Schult, 2020), which may have a larger impact on meaning in life after having children. Together, these studies suggest that the addition of children into a family has the potential to reorganize the central sources of meaning in life from what existed pre-parenthood, perhaps shifting the importance of the quality of one’s romantic relationship. The current study seeks to elucidate the potential moderating impact of the number of children on the association between relationship quality and meaning in life.

## The current study

Theorists assert ‘the idea that our social relationships directly influence the subjective meaningfulness of existence is incontrovertible’ (King and Hicks, 2021), yet existing evidence points to complexity in this association. The current study investigates the contribution of familial (i.e., romantic partner and children) relationships to self-reported meaning in life. Aim 1 investigates if the quality of an individual’s romantic relationship is associated with their level of meaning in life. First, we test the hypothesis that higher romantic relationship quality will be associated with greater levels of meaning in life (HO1a). Next, we test the hypothesis that relationship quality will be more strongly linked to meaning in life for women than men (HO1b). Aim 2 investigates the role of children in an individual’s level of meaning of life. We test the hypothesis that having more children will be associated with greater levels of meaning in life (HO2a). Given mixed literature, we hypothesize the number of children will be associated with meaning for both men and women at similar magnitudes (HO2b). Finally, in order to consider the intersectionality of individual identity and context in relation to meaning in life, we conduct an exploratory analysis examining a potential three-way interaction between relationship quality, number of children, and gender on meaning in life (HO3). Given the paucity of past research in this domain, we have limited literature from which to form hypotheses for this three-way interaction.

## Method

### Procedures and participants

Data were collected as part of a larger cross-sectional investigation of couples coping with COVID-19 between December 11, 2020, and February 11, 2021. Nationwide recruitment, consenting, and data collection of 504 individuals took place through the online platform Prolific. The University of Southern California Institutional Review Board approved the study. Eligibility criteria included being 18 or older, residing in the United States, and living with a romantic partner. Exclusion criteria for the current analyses included (a) failing 50% or more attention checks (*n =* 9), (b) not identifying as a woman or man as the sample size was too small to meaningfully detect effects for non-binary individuals (*n =* 3), (c) no longer living with or in a relationship with their partner (*n =* 6), or (d) having incomplete data (*n* = 13). A total of 473 individuals with complete data were included in the analytic sample (53.3% women; 100 couples) with ages ranging from 19 to 72 (*M* = 34.5, *SD* = 9.72). Participants were racially and ethnically diverse: 31.7% white, 22.4% Hispanic/Latino, 22.2% Asian, 20.7% Black, and 3.0% multiracial. Most participants were employed (87.5%) and had a BA degree or higher (67.9%). Participants had been cohabitating for an average of 8.2 years (*SD* = 7.6), 64.1% of the sample was married, and 4.4% of women and 4.5% of men were in same-sex relationships.

### Measures

#### Meaning in life

Six items assessed the amount of general meaning participants experienced in their lives, based on Martela and Steger’s (2016) trichotomy of meaning in life. One positively worded and one negatively worded item (reverse-coded) assessed each of the following dimensions: sense of purpose, coherence, and significance. Items were rated on a scale from 0 (‘Not at all’) to 3 (‘A lot’) and higher sum scores indicated more meaning (*α* = 0.91 for men and women).

#### Relationship quality

Relationship quality was assessed with the sum of the six-item Quality of Marriage Index (QMI; Norton, 1983), in which five items have a scale from 1 (‘Strongly disagree’) to 7 (‘Strongly agree’), and one has a scale from 1 (‘Extremely low’) to 10 (‘Extremely high’). Higher scores indicate higher relationship quality (α = 0.96 for men and 0.97 for women).

#### Number of children

All participants were asked to detail who was living in their home via open-ended response, including the age and relationship of the individual to the participant. Participant’s number of children under 18 living in the home was counted, and participants were categorized into three groups in order to create more balanced samples: no children, one child, and more than one child. Approximately half (55.0%) reported no children at the time of data collection, 20.5% had one child, and 24.5% had more than one child. Among the parents, the average number of children in the household was 1.66 (*SD* = 0.74) and ages of the children ranged from newborn to 18 years (*M* = 8.8, *SD* = 5.09).

#### Descriptives and covariates

Several multiple choice questions were created to assess demographic information such as employment status, educational attainment, and age. The degree of religiosity was measured using a single item, ‘Are you religious?’ on a scale from ‘Not at all’ (0) to ‘Extremely’ (6). General feelings of connectedness were assessed with the question, ‘How much have you been feeling connected to others?’ on a scale from ‘Not at all’ (0) to ‘A lot’ (3).

### Analytic approach

Descriptive results for study variables, including differences by gender and race/ethnicity, are presented first, as well as within-person correlations. The primary analysis consisted of hierarchical linear mixed modeling in R using *lme4* (Bates et al., 2015) to account for dependency of the dyadic data (i.e., some individual subjects were nested within couple dyads). We ran four models, each controlling for age, race/ethnicity, religiosity, and feelings of connectedness. In the first model, main effects of relationship quality, number of children (i.e., 0, 1, more than one), and gender, as well as covariates, were entered as predictors of meaning in life (HO1a and HO2a). Second, a model was constructed to test the interaction between relationship quality and gender, controlling for number of children and covariates (HO1b). Third, a parallel model tested the interaction between number of children and gender, controlling for relationship quality and previous covariates (HO2b). Fourth, we tested the exploratory three-way interaction between relationship quality, number of children, and gender on meaning in life (HO3). Significant interactions were decomposed using simple slope analyses. All continuous predictors were grand mean-centered and standardized for analyses. White individuals were treated as the reference group for race/ethnicity as they accounted for the largest portion of the sample.

## Results

### Descriptive analyses and bivariate correlations

Sociodemographic characteristics of the sample are presented in Table 1. Descriptive statistics and bivariate correlations are presented in Table 2. Men had greater meaning in life, feelings of connectedness, and were older compared to women. Meaning was positively correlated with relationship quality, number of children, religiosity and feelings of connectedness for both women and men. Meaning was positively associated with age for women, but not men. There was also a positive correlation between relationship quality and religiosity for men. Relationship quality was additionally positively associated with feelings of connectedness for both men and women. Number of children was positively linked to religiosity and age for men and women, and to feelings of connectedness for women.

**Table 1:** Sociodemographic characteristics by race.

	Total sample
*N*	473
Mean age in years (SD)	34.5 (9.7)
Gender (% male)	46.7
Education in %	
Some high school or completed high school	7.8
Some college	24.5
Bachelor’s degree	35.8
Additional schooling above a Bachelor’s	31.9
Employment status (before COVID-19) in %	
Full-time	74.7
Part-time	15.4
Unemployed	9.9
Individual annual income (before COVID-19) in %	
≤ $25,000	26.0
$25,000–$60,000	37.6
$60,000–$100,000	26.3
> $100,000	10.1

**Table 2:** Descriptive statistics and bivariate correlations among study variables.

	*M* (*SD*)		1	2	3	4	5	6
	Women	Men						
	(*n* = 252)	(*n* = 221)						
1. Meaning in life	12.57 (4.63)^a^	13.75 (4.39)^a^		0.23^***^	0.15^*^	0.33^**^	0.22^**^	0.18^**^
2. Relationship quality	37.89 (7.89)	38.39 (6.77)	0.41^***^		−0.12	0.12^*^	−0.01	−0.09
3. Number of children	0.73 (1.01)	0.76 (0.92)	0.21^**^	−0.06		0.13^*^	0.33^***^	0.01
4. Connectedness	1.19 (0.92)^b^	1.36 (0.89)^b^	0.34^***^	0.26^***^	0.10		0.19^**^	0.06
5. Religiosity	2.20 (2.19)	2.40 (2.16)	0.31^***^	0.18^**^	0.25^***^	0.17^**^		0.12
6. Age	34.18 (9.67)^c^	34.91 (9.79)^c^	0.10	−0.02	0.16^*^	−0.02	0.02	

Means and standard deviations are listed for each group (women and men). ^abc^Matching superscripts indicate significant gender differences. Pearson’s correlations for women are presented above the diagonal; correlations for men are presented below the diagonal. Number of children measures only children under 18 years of age. ^*^p < 0.05, ^**^p < 0.01, ^***^p < 0.001.

Results from a one-way between-subjects analysis of variance (ANOVA) indicated that meaning in life differed between the racial/ethnic groups (see Table 3). Tukey’s Honest Significant Difference (HSD) *post hoc* comparisons showed that Black participants reported more meaning than Asian (*p* = 0.02) and Hispanic/Latino (*p* = 0.005) participants. Relationship quality differed between the racial/ethnic groups, such that white participants reported greater relationship quality compared to Asian participants (*p* = 0.03). Parenting status also differed significantly by racial/ethnic groups, such that Asian participants had fewer children compared to white (*p* = 0.04) and Black (*p* < 0.001) participants. Similarly, Black participants reported significantly greater religiosity compared to white, Asian, and Hispanic/Latino participants (all *p*’s < 0.001). Black participants reported greater feelings of connection than Asian and Hispanic/Latino participants (both *p*’s = 0.02). Age did not differ by racial/ethnic group. Given these group differences, race/ethnicity was included as a covariate for all substantive analyses.

**Table 3:** Group differences in study variables by racial/ethnic group.

	White	Latino/Hispanic	Asian	Black	Multiracial	Test statistic	*p-*value
	(*n* = 150)	(*n* = 106)	(*n* = 105)	(*n* = 98)	(*n* = 14)		
	*M* (*SD*)						
Meaning in life	13.41 (4.28)	12.25^a^ (5.21)	12.50^b^ (4.46)	14.44^ab^ (3.92)	12.00 (5.05)	*F*(4, 468) = 3.96	0.004
Relationship quality	39.63 (6.46)^a^	37.86 (7.81)	36.93 (7.23)^a^	37.34 (8.29)	38.43 (6.05)	*F*(4, 468) = 2.59	0.036
Number of children	0.75^a^ (0.85)	0.66 (0.83)	0.46^ab^ (0.77)	0.92^b^ (0.85)	0.57 (0.85)	*F*(4, 468) = 4.27	0.002
Connectedness	1.24 (0.93)	1.16^a^ (1.00)	1.17^b^ (0.78)	1.55^ab^ (0.85)	1.14 (0.86)	*F*(4, 468) = 3.23	0.012
Religiosity	2.19 (2.24)^a^	1.87^b^ (2.10)	1.79^c^ (2.01)	3.39^abc^ (1.96)	2.71 (2.30)	*F*(4, 468) = 9.44	<0.001
Age	35.58 (9.45)	34.30 (10.38)	34.39 (10.36)	33.14 (8.42)	35.57 (11.07)	*F*(4, 468) = 9.96	0.409

^abc^Matching alphabetic superscripts indicate the significant mean differences in outcome variables between racial/ethnic groups.

### Inferential analyses

**Figure 1:**
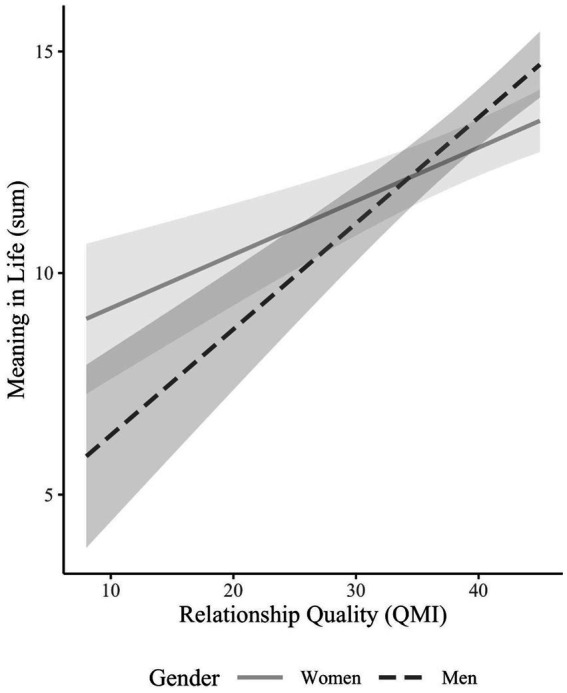
Interaction effects of relationship quality and gender on meaning in life.

**Figure 2:**
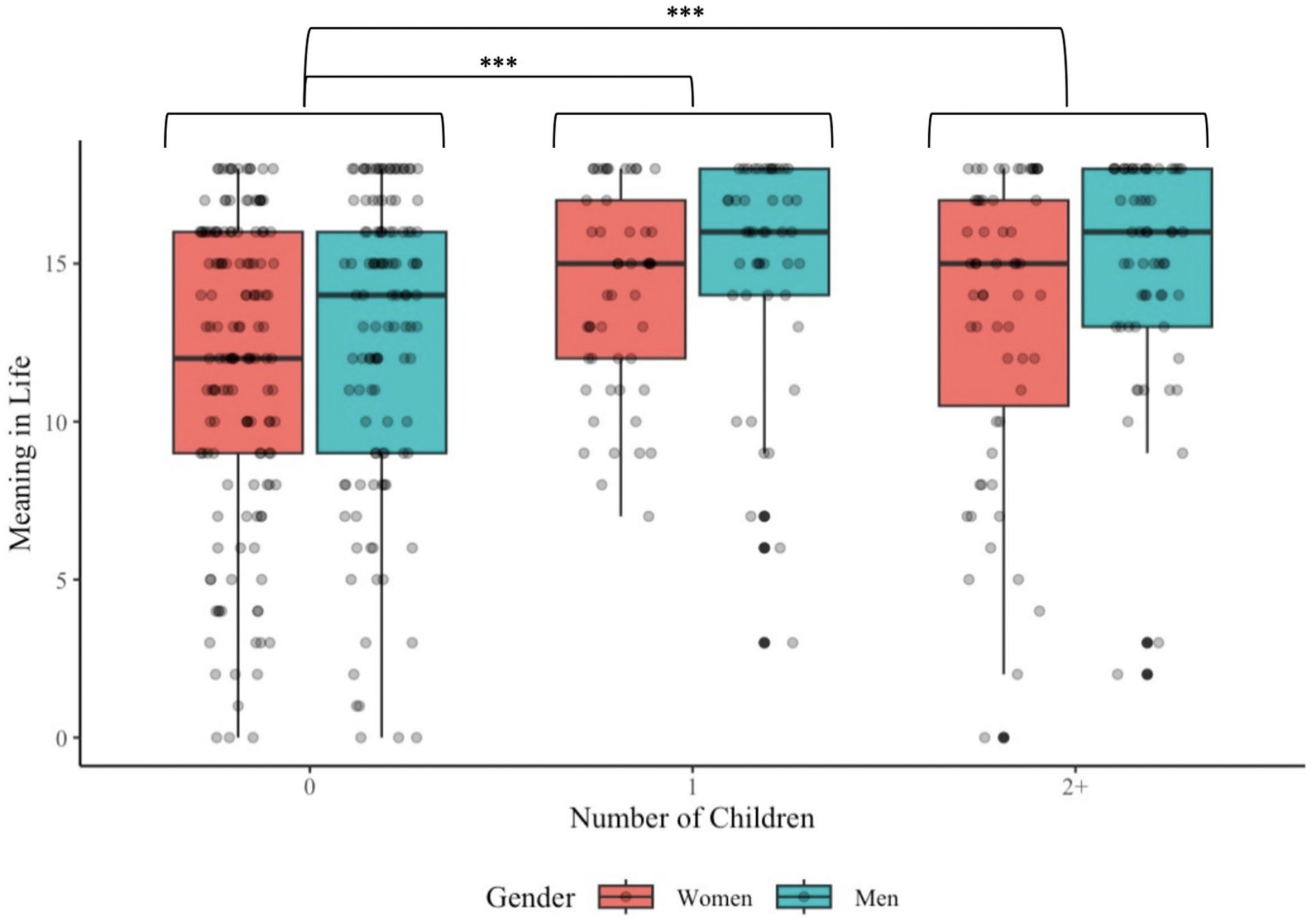
Group differences in meaning. Figure depicts data from the meaning in life measure, grouped based on number of children and gender. Significant differences emerged when comparing meaning in life between people with no children and people with one child, as well as between people with no children and people with more than one child. Although not depicted in the figure, men had higher meaning in life than women (*p* = 0.007). **p < 0.001.

Results for Aim 1 appear in the first and second columns of Table 4 and in Figure 1. Beyond the significant positive main effect of relationship quality on meaning in life (HO1a), a significant interaction with gender emerged. Simple slopes suggested the association between relationship quality and meaning was stronger for men (*B* = 0.39; SE = 0.06, *p* < 0.001) than women (*B* = 0.21; SE = 0.05, *p* < 0.001), counter to HO1b. The first and third columns in Table 4 and Figure 2 present results for Aim 2. There was a significant positive main effect for number of children on meaning in life (HO2a), but not an interaction with gender (HO2b). Figure 2 illustrates a follow up two-way ANOVA that compares the groups based on gender and number of children [*F*(2, 467) = 0.180, *p* = 0.84]. This revealed a main effect of gender (*p* = 0.007), such that men had higher meaning than women and a main effect of number of children (*p* < 0.001), such that people with one child or more than one child both had higher meaning than those with no children. The mean (SD) of meaning for women with 0, 1, or 2+ children was 11.71 (4.84), 14.10 (3.23), and 13.36 (4.70), whereas for men it was 12.71 (4.84), 14.98 (3.57), and 14.88 (3.45), respectively.

**Table 4:** Main and interactive effects between relationship quality, number of children, and gender on meaning in life.

Coefficient	Main effects		Aim 1 interaction		Aim 2 interaction		Exploratory model	
	*B*	*SE*	*B*	*SE*	*B*	*SE*	*B*	*SE*
Intercept	−0.11	0.09	−0.11	0.08	−0.11	0.08	−0.12	0.08
Age	0.15^***^	0.04	0.15^***^	0.04	0.15^***^	0.04	0.14^***^	0.04
Asian	0.08	0.11	0.10	0.11	0.08	0.11	0.11	0.11
Hispanic/Latino	−0.05	0.11	−0.04	0.11	−0.05	0.11	−0.04	0.11
Black	0.20	0.12	0.20	0.12	0.20	0.12	0.23	0.12
Multiracial	−0.22	0.23	−0.18	0.23	−0.22	0.23	−0.16	0.22
Religiosity	0.13^**^	0.04	0.12^**^	0.04	0.13^**^	0.04	0.12^**^	0.04
Connectedness	0.23^***^	0.04	0.22^***^	0.04	0.23^***^	0.04	0.22^***^	0.04
Gender	0.11	0.07	0.10	0.07	0.11	0.07	0.09	0.07
Relationship quality	0.27^***^	**0.04**	0.21^***^	0.05	0.27^***^	0.04	0.20^***^	0.05
Number of children	0.13^**^	**0.05**	0.13^**^	0.05	0.13^*^	0.06	0.12^*^	0.06
Relationship quality x Gender			0.18^*^	**0.07**			0.22^**^	0.07
Number of children x Gender					**0.00**	**0.07**	0.04	0.07
Relationship quality x Number of children							0.00	0.05
Relationship quality x Number of children x Gender							−0.17^*^	**0.08**
Random effects								
σ^2^	0.42		0.38		0.42		0.37	
ICC	0.43		0.48		0.43		0.49	
Observations	473		473		473		473	
Marginal R^2^/Conditional R^2^	0.265/0.581		0.273/0.620		0.265/0.581		0.279/0.633	

^*^p < 0.05, ^**^p < 0.01, ^***^p < 0.001. Number of children measures only children under 18 years of age. Bolded numbers represent the relevant statistics for each aim.

**Figure 3:**
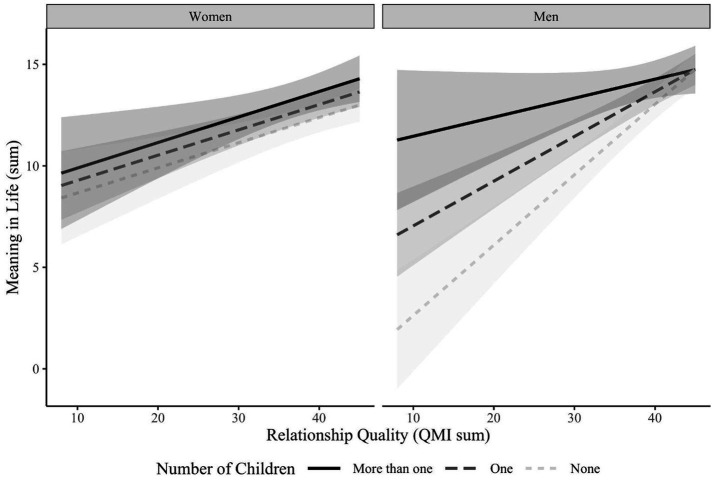
Interaction effects of relationship quality, number of children, and gender on meaning in life.

The exploratory Aim 3 model, presented in the fourth column of Table 4, showed a significant three-way interaction between relationship quality, number of children, and gender. Figure 3 plots this interaction. Simple slope analyses revealed significant and similar associations between relationship quality and meaning in life for women with no children (*B* = 0.20; SE = 0.07, *p* < 0.001), one child (*B* = 0.20; SE = 0.05, *p* < 0.001), and more than one child (*B* = 0.20; SE = 0.09, *p* = 0.02). For men, relationship quality was positively associated with meaning in life among men with no children (*B* = 0.56; SE = 0.09, *p* < 0.001) or one child (*B* = 0.36; SE = 0.06, *p* < 0.001), but not for men who had more than one child (*B* = 0.15; SE = 0.11, *p* = 0.16).

In all tested models, the covariates of age, level of religiosity, and feelings of connectedness were each positively associated with meaning in life. The fixed (i.e., within-person) effects in each of the models explained over 26% of the variance in meaning in life and the total models (fixed and random effects) explained over 58% of the variance.

### Supplementary analyses

Given the differences in variables of interest by racial/ethnic group, we conducted supplementary analyses to investigate if racial/ethnic group was a relevant factor in the two-way interaction between relationship quality and gender on meaning in life. However, no significant three-way interactions emerged between relationship quality, gender, and racial/ethnic group (all *p-*values >0.05). Further, no significant three-way interactions emerged when considering interactions between relationship quality, gender, and education level or employment status.

## Discussion

In this study, we investigated associations between romantic relationship quality, number of children, and gender on meaning in life in a diverse sample during the COVID-19 pandemic. We found that, above and beyond well-established sources of meaning such as religiosity and feeling connected to others, the quality of the relationship with a romantic partner was associated with higher meaning in life for men and women, though more strongly for men. In addition, the significant positive link between number of children and meaning in life for both genders adds to a growing body of literature that highlights how parenthood enhances meaning in life. However, nuance emerged when considering the role that gender plays in the contributions of relationship quality and children to meaning in life. For men, the strength of the association between relationship quality and meaning in life was lower when they had one child compared to no children, and the association failed to reach significance for men with more than one child. For women, relationship quality was associated with meaning in life at essentially the same magnitude regardless of number of children. We also replicated past studies that showed a positive main effect of religiosity and feelings of connectedness on meaning in life. It is notable that our models explain a high level of variability in meaning in life, which supports the theoretical assertion that social relationships incontrovertibly are associated with meaning in life, although the direction of effects cannot be fully ascertained.

Our findings supported our hypotheses about the importance of both romantic relationship quality and children for an individual’s sense of meaning in life, perhaps by way of adding to an individual’s sense of purpose, coherence, and/or significance (Morse and Steger, 2019). Further, high-quality relationships and the parenting role may both enhance meaning due to their likely influence on ‘mattering’ a recently highlighted element of meaning in life (George and Park, 2016). The literature on ‘mattering’ suggests that ‘leaving a legacy that will transcend one’s self’ is the strongest contributor to judgments of meaning in life (Costin and Vignoles, 2020). While there are many alternative ways to leave a legacy (e.g., mentoring, teaching, career contributions, volunteering), our results underscore that family life is a rich environment in which the connection to others and feeling connected to something larger than oneself may be particularly salient or meaningful.

Although we hypothesized that relationship quality would be more strongly linked to meaning for women than men, our results suggest the opposite pattern. One possible explanation that helps make sense of why romantic relationship quality was more strongly associated with meaning in life for men than women comes from prior work demonstrating that heterosexual women tend to have a broader range of close personal relationships than do heterosexual men. Although prior research does not show gender differences in overall number of social relationships (Wrzus et al., 2013), women tend to have higher quality social support (Rosenfield and Mouzon, 2013), less social isolation (Röhr et al., 2022), more confidants (McPherson et al., 2006), and are less likely to name their partner as their closest relationship (Fuhrer and Stansfeld, 2002). Men more frequently turn to their romantic partners for social support, whereas women more often turn to other women (Taylor et al., 2000). It follows that for women, the strength of the association between the level of relationship quality and meaning in life is weaker than for men perhaps because women likely have greater sources of high-quality, close relationships—beyond their romantic partner—that contribute to meaning in life.

Our results do not support a gendered difference in the way that having children contributes to meaning in life. This result is in line with Musick et al. (2016), but counter to findings from studies that consider a broader range of parenting elements, such as time spent caregiving, in a far larger sample (e.g., Nelson-Coffey et al., 2019). Beyond the number of children, there are several aspects of parenting (e.g., parenting stress, childcare responsibilities, parent–child bonding, child temperament) that we did not consider in this study. Future research would benefit from investigating a more nuanced picture of the contribution of parenting to meaning, such as considerations of gender differences at different parenting stages, non-binary and gender-fluid parents, broader family contexts, and mechanisms by which parenthood confers meaning.

Our three-way interaction indicates that having more children decreases the strength of the association between relationship quality and meaning for men, but not women. These results show the complexity by which social relationships impact meaning in life—that is, for men with additional children, there seems to be a downward shift in the impact of the romantic relationship on meaning in life. While it is a reasonable assumption that each additional child in a family changes family dynamics in ways that have downstream impacts on meaning in life for parents, more work is necessary to better understand these patterns. Although men overall show stronger links between quality of their romantic relationship and meaning, having children may increase the number of deep, meaningful connections for men such that their romantic relationships subsume a less prominent role in their experience of meaning. This is in contrast to men with no children where the quality of the romantic relationship is highly linked to meaning; men without children in low quality romantic relationships report notably low meaning. Another possible explanation may relate to the type of parenting each gender performs and an individual’s resulting sense of purpose. Mothers tend to take on responsibilities related to childcare —tasks like feeding, bathing, and emotional support (Sullivan, 2019), as well as needs such as play (Yeung et al., 2001). It is possible that the time burden (Kohler, 2012) and the stressors of parenting related to these tasks compromise a sense of meaning in mothers (Taubman-Ben-Ari et al., 2021). Moreover, in traditionally gendered households, fathers tend to play the role of a protector in their family (Yaffe, 2020). Taken together, parenthood may confer the identity of the ‘provider’ to men, which would likely offer a greater sense of purpose. It is possible that as the size of a man’s family increases, his romantic partner becomes subjectively subsumed within the family unit for whom the man ‘provides.’ In turn, the *quality* of the romantic relationship may begin to matter less for the father’s sense of meaning in life. For women, on the other hand, the role of the mother tends to be functionally distinct from her experience as a romantic partner, and thus the association between relationship quality and meaning in life is not affected by the presence or number of children for women. This is just one of many possible explanations for the exploratory finding, and future research should measure how couples view their role as a parent, including how important the parenting role is to their self-identity, as this may help explain contributions to their meaning in life (Thoits, 2012).

Despite its contributions, this study is not without limitations. First, data from this study were collected only on adults in romantic relationships. As such, the conclusions from the study apply only to partnered individuals who identified either as men or women, as we could not compare differences in meaning in life between partnered and unpartnered individuals. Future research is critical to better understand meaning in individuals who are non-partnered, both those with and without children. Individuals without children or romantic partners can find meaning through other social relationships including friends and family, or still engage in generative behaviors that serve others. Second, we also only included individuals who identified either as men or women and thus we could not consider group differences by other types of gender identification (e.g., non-binary). Third, the distribution of number of children was skewed. More than half (55%) of the sample did not have children, creating an overrepresentation of non-parents in our analyses compared national estimates (40%, U.S. Census Bureau, 2021). Further, a total of 29 (6.1%) study participants were living with at least one adult child (age 18 or older), and were thus categorized by our study as having no children due to our age cut-off of 18. There is great heterogeneity in the reasons an adult child may be living with their parents (e.g., caregiving for a parent, cultural norms, financial difficulties during the pandemic causing families to combine households). Future studies should investigate the impact of living with an adult child on the meaning in life of parents. Fourth, the cross-sectional nature of this investigation compromises our ability to make causal conclusions about relationships and meaning or test the direction of effects. Fifth, and relatedly, selection effects cannot be ruled out, such that people with higher meaning may select into more satisfying relationships, or people with higher meaning in life are more likely to choose to have children. Individuals in the present study overall had high meaning in life, and people characterized by higher meaning may view their relationships with partners and/or children differently than people with low meaning.

Sixth, the current study collected data approximately one year into the pandemic, when COVID-related restrictions and changes still greatly impacted people’s social contexts. The importance of various sources of meaning likely ebbed and flowed across the course of the pandemic as COVID-19 may have altered individuals’ perspectives on ‘what really matters’ in their lives (Baños et al., 2023). Moreover, circumstances surrounding the pandemic likely intensified the importance of home relationships as those close relationships (e.g., partners and children) made up more of people’s social world at that time. However, the COVID-19 pandemic can still be used as an informative proxy for other situations that threaten someone’s well-being, or for circumstances that draw into focus questions about meaning in life. Sixth, there are numerous other sources of meaning in life (e.g., community activities, personal development; Weinstein et al., 2012) that we did not investigate here. Individuals with or without children or romantic partners find meaning through other social relationships including friends, family, co-workers, or through actions that serve others or benefit society more generally. For example, investing in one’s community, engaging in spiritual or creative endeavors, practicing gratitude, or demonstrating acts of kindness may be equally impactful for meaning and do not rely on a romantic partner or children (Bono and Sender, 2018; Corbett, 2018). Future research on meaning in life may benefit from assessing associations between a wider range of social relationships and activities.

## Conclusion

Researchers are still beginning to understand how different sources of meaning interact with one another and uncover the complexity with which people’s general meaning in life is impacted at different life stages, or in different contexts (e.g., pandemic). This study fills an important gap by assessing multiple potential sources of social contributions to meaning (as specifically called for by O’Donnell et al., 2014), and thereby has implications for theoretical perspectives on meaning in life. Future policy can also benefit from considering the results presented here. For example, our data supports the need for more inclusive family leave policies that would enable mothers and fathers to spend more time with their children which could improve their overall well-being. Finally, the presented results have implications for improvement of meaning in life and related therapeutic interventions. Although research has found brief mindfulness and narrative approaches to be successful at improving meaning in life (Manco and Hamby, 2021), our findings additionally implicate relationships as important for meaning. Just as work suggests that meaning-focused interventions with couples are an important potential way to improve relationship functioning (Schulenberg et al., 2010), our results suggest the reverse may also be true. That is, therapeutic work that aims to bolster a couple’s relationship or to highlight an individual’s familial relationships would likely improve individuals’ sense of meaning. In light of this, therapeutic interventions that highlight connections between dyadic functioning and values (e.g., ACT-informed work on values guided actions) may serve to benefit the couple relationship, parenting, and individual well-being. This investigation thus emphasizes the need for future studies that measure various sources of meaning in life, multiple elements of meaning, and the ways that people perceive and value their social roles.

## Author’s note

This study was preregistered with Open Science Framewok (Margolin et al., 2020, https://doi.org/10.17605/OSF.IO/XWD5C).

## Data availability statement

The raw data supporting the conclusions of this article will be made available by the authors, without undue reservation.

## Ethics statement

The studies involving humans were approved by University of Southern California Institutional Review Board. The studies were conducted in accordance with the local legislation and institutional requirements. The participants provided their written informed consent to participate in this study.

## Author contributions

AG: Conceptualization, Formal analysis, Visualization, Writing – original draft, Data curation, Methodology. YR: Investigation, Visualization, Writing – original draft, Writing – review & editing, Formal analysis, Methodology. EA: Writing – original draft, Writing – review & editing. GC: Conceptualization, Writing – review & editing, Methodology. HR: Conceptualization, Data curation, Writing – review & editing, Project administration. YK: Data curation, Writing – review & editing, Project administration. GM: Conceptualization, Funding acquisition, Supervision, Writing – review & editing, Methodology, Project administration.
